# Who Is at Risk? Population Characterization of Alcohol Self-Administration in Nonhuman Primates Helps Identify Pathways to Dependence

**Published:** 2008

**Authors:** Kathleen A. Grant, James Stafford, Allison Thiede, Caitlin Kiley, Misa Odagiri, Betsy Ferguson

**Keywords:** Monkey, alcoholism, self-administration, behavior, risk, genetics

## Abstract

Alcohol abuse and dependence are human conditions for which no full equivalent exists in animals. Nevertheless, animal models frequently are used to study various aspects of alcohol dependence that cannot be easily or ethically assessed in humans, including neurobiological mechanisms underlying alcohol dependence. Many of these animal models involve rodents; however, the characteristics (i.e., phenotypes) of chronic heavy drinking may be limited in these species. Nonhuman primates add an important translational aspect to the study of alcohol abuse and alcoholism. Their genetic, anatomical, physiological, and behavioral similarity to humans offers unique opportunities for identifying risk factors that may predispose a person to or accelerate the course of alcohol addiction. Studying alcohol consumption in nonhuman primates, including the distribution of drinking levels in a population, also can be uniquely informative to alcohol research. For example, research on the self-administration procedures in primates can help scientists identify risk factors for excessive alcohol consumption in humans. The phenotype of excessive drinking then can serve as the starting point to test and verify the underlying genetic and environmental influences. The resulting findings, in turn, can help guide prevention and treatment strategies.

Alcohol addiction—or alcohol dependence—is a chronic and progressive disorder that has a significant detrimental impact on the drinker, his or her family and community, and society as a whole. Accordingly, it is important to identify the mechanisms contributing to the development of alcohol dependence as well as the factors that increase an individual’s risk of becoming alcohol dependent. Only with this knowledge will researchers and clinicians be able to develop new treatment approaches and effective interventions to reduce or prevent the development of alcohol problems in people at risk. One important step is to identify the neurobiological mechanisms that are affected by alcohol use and/or which drive alcohol use and the progression to dependence. Although alcohol dependence is a uniquely human disease that does not occur naturally in animals, animal models frequently are used to study various aspects of the development of alcohol dependence and its consequences because the corresponding experiments would not be feasible or ethical to conduct in humans. This is particularly true in the area of neuroscience, where direct analyses of brain pathways are limited in living individuals (e.g., measurements using imaging methods that reflect alcohol-induced changes in brain function).

Most animal models of alcohol dependence involve rodents, which are easy to obtain in sufficiently large numbers and also have short generation times, so that the effects of excessive alcohol exposure can be rapidly determined. However, the lifespan and stages of development, the neuroanatomical and physiological complexity, and the social behavior of rodents and humans differ to such an extent that not all aspects of the human condition can be adequately modeled or are sufficiently transferable to humans. Nonhuman primates, in contrast, although lacking in a number of ways, offer researchers the ability to model aspects of the human condition more closely.

Monkeys and apes^1^ (i.e., nonhuman primates) have a rich history as experimental animals in the study of biomedical disease processes (http://www.primate.wisc.edu/pin/) because they are most similar to humans in that they have relatively long lifespans, go through parallel developmental stages, and share similar genetic predispositions. The value of nonhuman primate studies is particularly evident in research aimed at assessing the risk of developing behavioral disorders, because like humans, nonhuman primates experience complex social and affective processes. The basic data generated by studies of nonhuman primates then can be followed with experimental designs that address the underlying mechanisms of the disorder under investigation and can be the basis for the development of targeted prevention and therapy.

Nonhuman primates also have been used to model aspects of alcohol abuse and dependence, such as mechanisms underlying alcohol-related organ damage. In addition, a large body of research has examined the neurobiological basis for and consequences of alcohol use, including neurobiological adaptations hypothesized to mediate addictive behaviors associated with alcoholism (Barr and Goldman 2006; Grant and Bennett 2003). For example, as described later in this article, studies in nonhuman primates have helped elucidate the interactions between alcohol and various brain-signaling pathways (i.e., neurotransmitter systems).

As with rodent subjects, most studies that use nonhuman primates as subjects involve passive administration of alcohol (chemically known as ethanol) to study the consequences of heavy drinking—that is, the alcohol is administered by the experimenter. However, monkeys also have been used to model human alcohol consumption itself by using procedures that typically incorporate access conditions in which the monkeys themselves initiate alcohol intake. This is known as self-administration.

Using such procedures, it is possible to directly identify risk factors for abusive alcohol drinking. Commonly acknowledged biological factors that put an individual at risk of developing excessive ethanol intake include gender, age of onset, metabolism, response to stress, impulsivity, family history of alcoholism, and sensitivity of the brain reward pathway. These factors are independent of, but interact with, environmental risk factors, such as cost, alcohol availability, and social acceptability of drinking. Determining the risk factors for developing chronically high alcohol intake, as well as determining which risk factors predict specific subtypes of alcoholism, are critical objectives for improving public health policy, prevention, and treatment.

One important step in that direction is to determine the population distribution of ethanol drinking and to characterize the biological traits of the individuals at either end of the drinking distribution (i.e., of individuals with particularly low or particularly high levels of alcohol consumption). This article reviews the findings of studies that have assessed the drinking distribution in monkey populations and have investigated the acquisition of drinking behavior in these animals. The article also explores how information on the population distribution of drinking can help investigators identify the neurological basis for alcohol intake levels and patterns.

## Distribution of Alcohol Consumption in Monkey Populations

**Figure 1:**
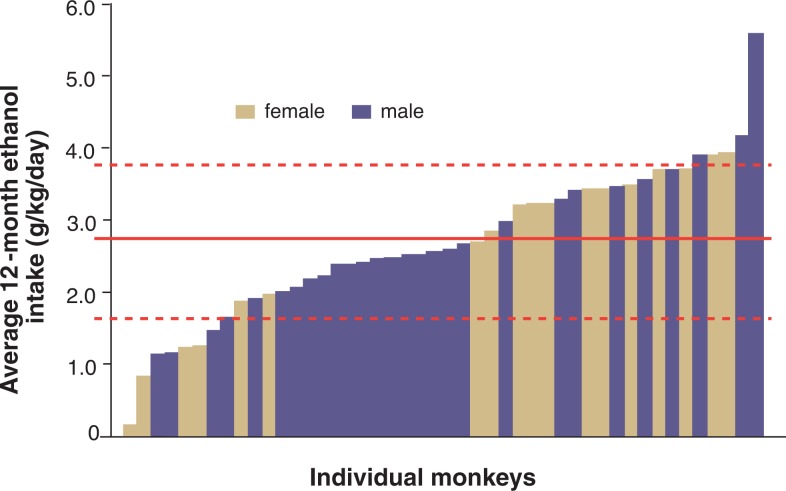
Distribution in a population of cynomolgus monkeys (*n* = 46) of average daily ethanol intake in grams per kilogram body weight per day (g/kg/day) over a 12-month period, during which the animals had access to 4 percent ethanol for 22 hours per day. The population was composed of four separate cohorts of monkeys studied in groups of 10 to 12 per cohort (for details, see Grant et al. 2008; Vivian et al. 2001). The red line at 2.7 g/kg/day indicates the mean value for the population and dashed lines indicate 1 standard deviation (1.03 g/kg/day) from the mean.

With the growing number of alcohol self-administration studies using monkeys, it is becoming increasingly evident that the individual differences in alcohol consumption found in humans also exist in nonhuman primates (see figure 1). Thus, only a proportion of either monkeys or humans exposed to alcohol develop chronically high intake patterns (Dawson 2000*a*; Grant et al. 2008; Vivian et al. 1999, 2001). For example, approximately 80 percent of all the alcohol sold in the United States is consumed by only about 20 percent of all actively drinking adults (Dawson 2000*a*).

**Figure 2:**
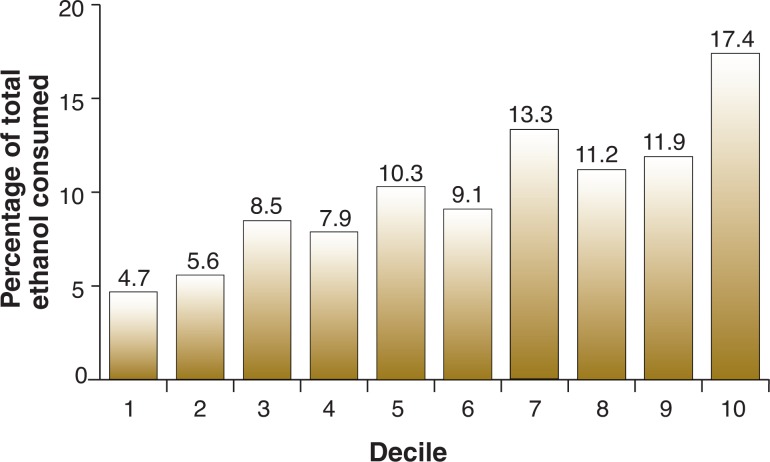
Percentage of total ethanol consumed by the population of adult male and female cynomolgus macaque monkeys (*n* = 46) shown in figure 1 and divided into deciles (the exact percentages of ethanol consumed by each decile of the population are given above the respective bar). Ethanol was consumed in a self-administration procedure that allowed access to ethanol for 22 hours/day, 7 days/week, for approximately 52 weeks (see caption for figure 1).

In monkeys, differences in the levels of alcohol consumption also exist, although the distribution is not as skewed as in humans. Thus, among cynomolgus macaque monkeys (*Macaca fascicularis*) living individually and given almost continuous access to ethanol (22 hours/day), the top 20 percent of drinkers consume approximately 30 percent of all alcohol drunk over a 12-month period (see figure 2). Conversely, the bottom 20 percent of drinkers consume only 10 percent of the total alcohol consumed. Overall, these findings suggest that cynomolgus monkeys exhibit fewer individual differences in alcohol consumption compared with humans.

One should note, however, that these studies represent extremely conducive drinking environments. That is, in the self-administration experiments described above, ethanol was easily available and the possibilities of negative outcomes attributed to intoxication generally were limited to personal health. For example, in studies using individually housed monkeys as subjects, there is a low response requirement for ethanol (i.e., the cost per drink is low), and social constraints that may limit drinking to intoxication, such as allocation of other resources (e.g., food, shelter, or health care), are not threatened by ethanol consumption. In general, these population statistics show that ethanol self-administration under relatively open-access conditions is a quantitative trait and perhaps reflects an estimate of the “biological vulnerability” of Old World primates, including humans, to repeated consumption of intoxicating levels of alcohol.

### Blood Ethanol Concentrations As a Measure of Alcohol Intake

Within both human and monkey populations, there clearly are some individuals who drink alcohol excessively. Before findings on these individuals in monkey populations can be translated into meaningful information on which humans are at particularly high risk of excessive drinking, researchers must select appropriate criteria for categorizing drinking as excessive. Indeed, there is an active debate among researchers conducting human epidemiological studies over how to measure hazardous consumption (Dawson 2000*b*, 2003; Greenfield and Kerr 2008). In nonhuman primate investigations, excessive drinking traditionally is defined only within the context of that study (Ervin et al. 1990; Grant and Johanson 1988; Vivian et al. 1999, 2001). Accordingly, alcohol consumption that is labeled excessive in one study in many cases may not approach the levels of consumption considered problematic in humans and may vary significantly from what is identified as high consumption in other nonhuman primate studies. For example, one study (Higley et al. 1991) defines excessive drinking as an average intake of approximately 1.0 g ethanol/kg body weight per day (g/kg/day), a level that other laboratories have defined as light (Vivian et al. 2001).

One approach to reconcile these inconsistencies in terminology is to focus on the frequency of voluntary alcohol self-administration to the point of intoxication, with intoxication defined as having a blood ethanol concentration (BEC) above 80 mg/dl (or 0.08 percent^2^) (Grant et al. 2008; Shelton and Grant 2001; Vivian et al. 2001). By measuring repeated self-intoxication, researchers can initiate cross-species comparisons of alcohol self-administration so that cumulative datasets from different laboratories can be assembled and broader conclusions can be drawn across all primate species studied. For example, by correlating the intakes of baboons and cynomolgus monkeys self-administering a matching concentration of ethanol in water with BECs (Grant et al. 2008; Kaminiski et al. 2008), investigators from different laboratories could equate the large differences in volumes of intakes, which reflect the three- to five-fold differences in body weight between cynomolgus monkeys and baboons.

**Figure 3:**
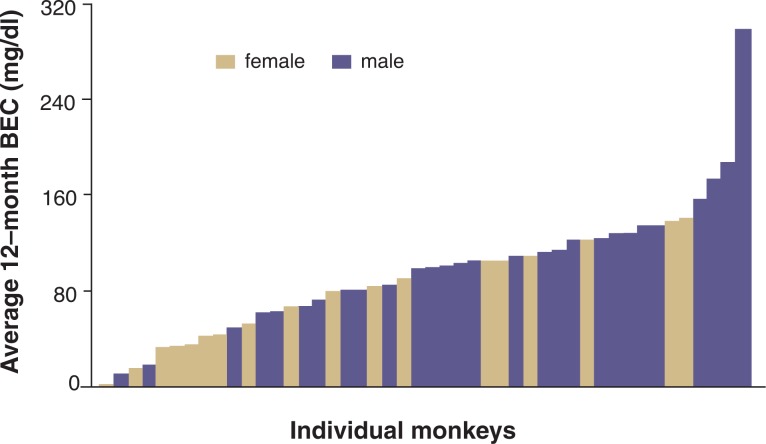
Distribution of average blood ethanol concentrations (BECs) among the monkeys described in figure 1, as determined during the 12 months of ethanol self-administration. BECs were determined from samples taken every fifth session from 46 monkeys; a total of 2,880 samples are represented here. Approximately 62 percent of the population had average BECs of 80 mg/dl or greater, suggesting that they regularly (daily) drank to intoxication. Males were more likely to drink to higher average BECs compared with females, likely reflecting their pattern of consumption during the day (see text). Differences in the distributions between the BECs shown here and the daily intakes shown in figure 1 are attributed to the residual ethanol intake after the blood samples were taken (i.e., nighttime and morning consumption).

**Figure 4:**
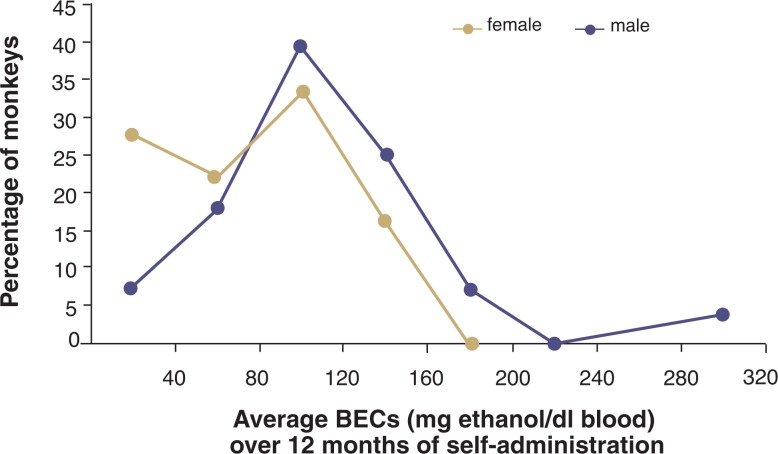
Frequency distribution of average blood ethanol concentrations (BECs) that encompass the entire range of average BECs for this population of monkeys (see figures 1 and 3 legends). For each monkey, 60 to 72 samples were taken over the entire period. Note that 50 percent of the females, but 75 percent of the males, had an average BEC over 0.08 percent (which is the proposed definition of intoxication).

A population distribution of BECs in the male and female cynomolgus monkeys from the alcohol intakes shown in figure 1 is illustrated in figure 3. The BECs were measured every fifth session for 12 months of 22-hours/day access to ethanol and water, with measurements taken between 6 and 7 hours after the start of the daily session and just before the lights went out in the room for the night (for details on the daily access schedule, see Grant et al. 2008).^3^ A comparison of figures 1 and 3 indicates that large individual differences in both average alcohol intake and BEC exist; however, gender differences are more apparent in the BEC distribution (figure 3) than in the daily intake distribution (figure 1). Specifically, half of the female monkeys tended to drink in a pattern resulting in BECs lower than 0.08 percent, whereas only 25 percent of the male monkeys appeared to limit their intakes so that BECs remained below 0.08 percent (see figure 4). These absolute consumption levels seen in the monkeys are higher than what has been found in humans (e.g., in the U.S. adult population for both sexes). However, the finding of higher average BECs in male monkeys is consistent with the observation that the 12-month prevalence rates for alcohol abuse according to the criteria established in the *Diagnostic and Statistical Manual of Mental Disorders*, *4th Edition* (DSM–IV) (American Psychiatric Association 1994) are higher in men than in women (gender ratios of 2.72 and 2.34 for alcohol abuse and dependence, respectively) (Grant et al. 2004). These gender differences also are seen when the population is broken down into subgroups based on race and age (Grant et al. 2004). Thus, the tendency for females to drink in a pattern that lowers risk for adverse outcomes (which is the primary diagnostic criterion of the DSM–IV classification) appears similar in macaque monkeys and humans.

Although BECs below 0.08 percent are associated with many functional consequences, including effects on subjective feeling, performance, learning, and physiological processes, the diagnosis of alcohol use disorders (AUDs) (which include alcohol abuse and alcohol dependence) specifically refers to the negative consequences of repeated self-intoxication (Grant et al. 2004). These criteria are met by approximately 5 percent of the U.S. adult population, for whom excessive alcohol intake is associated with negative personal and biomedical outcomes^4^ (Grant et al. 2004).

In comparison, approximately 62 percent of the macaque population mentioned above repeatedly engages in a daily drinking pattern that results in BECs above 0.08 percent (see figure 3) as well as negative biomedical outcomes. Specifically, monkeys with a daily intake of more than 3.0 g ethanol/kg body weight (g/kg) and resulting BECs above 0.08 percent (Vivian et al. 2001) show signs of brain dysfunction (Anderson et al. 2007; Budygin et al. 2003; Carden et al. 2006; Floyd et al. 2004; Hemby et al. 2006) as well as liver dysfunction (Ivester et al. 2007). Thus, like the finding of higher absolute alcohol consumption among the monkeys than among humans, this evaluation of ethanol self-administration demonstrates that a much greater proportion of monkeys can be labeled excessive drinkers (i.e., 39 percent as determined based on daily intakes above 3.0 g/kg or 62 percent as determined based on attaining BECs of more than 0.08 percent on a regular basis) compared with the proportion of humans (i.e., U.S. adults) who are diagnosed with an AUD (i.e., approximately 5 percent). This high level of alcohol consumption in the monkeys most likely reflects a biological predisposition to excessive alcohol drinking in both human and nonhuman primates under circumstances where other controls on behavior (e.g., social constraints and resource allocation) are not operative.

## Acquisition of Alcohol Drinking Behavior in Nonhuman Primates

Repeatedly engaging in self-intoxication is a learned phenomenon, and evidence suggests that humans and animals undergo an acquisition phase of varying length as they “learn to drink alcohol” (Samson and Grant 1990). Additional studies (for a review, see Grant and Bennett 2003) found that simple access to alcohol solutions generally is not sufficient to produce sustained self-administration of intoxicating quantities of ethanol in monkeys. In fact, most monkeys show an aversion to high concentrations (greater than 8 percent volume for volume [v/v]) of ethanol; however, some monkeys readily consume intoxicating quantities of less concentrated ethanol solutions (i.e., 5 percent v/v). Additional studies (Macenski and Meisch 1992) found that monkeys can acquire ethanol self-administration even if no specific induction procedure or alcohol with added flavorants is used. In these animals, however, the average intake is relatively low (i.e., 0.2 to 1.0 g/kg per 3-hour session, which corresponds to less than one to four drinks every 3 hours).

To produce elevated and consistent ethanol consumption, researchers commonly use specific initiation procedures (see Grant and Bennet 2003; Katner et al. 2004, 2007; Weed et al. 2008). The most common methods used to induce oral ethanol consumption in monkeys have been to deprive the animals of food, to flavor the alcohol solution with a preferred taste (e.g., fruit juice), or to use a schedule-induction procedure (Katner at al. 2007; Grant and Bennett 2003). With such induction procedures, ethanol intakes increase to over 1.0 g/kg/hour or the equivalent of four drinks per hour in rhesus monkeys (Macenski and Meisch 1992; Rodefer et al. 1999; Vivian et al. 2001; Williams et al. 1998) and in baboons (Weerts et al. 2006) during limited access (less than 4 hours/day).

Studies have not directly determined the efficacy of different induction procedures for establishing excessive or heavy-drinking outcomes. Still, there appears to be a consensus that once oral self-administration of ethanol has been established, “…the subsequent pattern and amount of drug intake appears to be independent of the acquisition procedure” (Meisch 2001, p. 119). Direct studies of the efficacy of induction procedures as well as examination of self-administration under the same access conditions following different induction procedures are needed and could be uniquely informative for assessing the risk of progression to heavy drinking in humans based on how alcohol use was initiated.

### Progression of Alcohol Consumption Following Induction

In general, studies have suggested that following induction, daily alcohol intake can be elevated by increasing the amount of time that ethanol is available. For example, following a certain induction procedure, cynomolgus macaques that received access to alcohol for a total of 30 minutes/day had an average intake of 1.0 to 1.25 g/kg/day (Shelton and Grant 2001), whereas animals that had been subjected to the same induction procedure but had alcohol access for 22 hours/day had an average alcohol intake of 2.7 g/kg/day (see figure 1). Daily alcohol intake also can be increased by imposing multiple, discrete sessions of self-administration during a 24-hour period (Katner et al. 2007). A combination of various measures also can increase alcohol intake. For example, using slight food deprivation, orange-flavored vehicle, and four discrete drinking sessions per day, Weed and colleagues (2008) demonstrated a high daily ethanol intake (mean intake of 4.6 g/kg) in pig-tailed macaques (*Macaca nemestrina*). Similar results have been reported in rhesus monkeys given flavored ethanol in two distinct 1-hour sessions per day (Katner et al. 2007).

When one contrasts the study parameters of the various induction procedures, it becomes clear that the outcomes reflect the intentions of the studies. The parameters of flavored alcohol solutions and discrete times of ethanol availability were designed to reduce variability between different animals in a study while increasing overall ethanol intake in a relatively short period of time (see Katner et al. 2007; Weed et al. 2008). This is a useful approach for investigating the progression of deleterious effects associated with high-dose alcohol intake (Katner et al. 2007; Weed et al. 2008) as well as ethanol reinforcement^5^ or the effects of potential pharmacotherapies (e.g., Katner et al. 2004; Meisch 2001; Shelton et al. 2001; Weerts et al. 2006).

In contrast, studies using the parameters of continuous 22-hour access and unflavored ethanol solutions result in wide individual differences in average daily ethanol consumption among the animals (i.e., in higher between-subject variability) (see figure 1). This approach particularly is useful for investigating who is at risk for heavy drinking. Based on the resulting population distribution (see figures 1 and 3), additional experiments can be designed to explore which predisposing organismal variables (i.e., genetic, physiological, and gender- and age-related factors) and environmental variables (i.e., stress and trauma, social organization, and alternative reinforcers) are associated with either end of the drinking spectrum.

The varied induction procedures and self-administration variables (e.g., use of nonflavored or flavored alcohol, time of day and duration of access, free alcohol availability versus operant responding,^6^ feeding conditions, etc.) make it difficult to compare the alcohol intakes of animals between studies. Thus, induction procedures differing from the ones leading to the population distributions shown in figures 1 and 3 (see Grant et al. 2008; Vivian et al. 2001) may result in different distributions.

Additionally, studies currently are underway to assess species differences in the population distribution of alcohol intake by using the same induction parameters and self-administration parameters for rhesus monkeys (*Macaca mulatta*) and cynomolgus monkeys. However, by documenting a population distribution of daily drinking averages as shown in figure 1 and using equipment that records each instance, duration, and amount of the alcohol solution that an animal drinks, researchers may be able to identify the precise drinking patterns that underlie various daily averages. Such analyses of alcohol intake patterns can help inform studies in humans aimed at validating alcohol measurement methodology, particularly in extrapolating between intake patterns and BECs (see Grant et al. 2008).

## Identifying the Neurobiological Basis for Alcohol Intake Levels and Patterns Using Population Distributions

Population studies such as the ones described above also can benefit neuro-science research aimed at understanding a variety of aspects of AUDs, including the role of tolerance, modification of the central nervous system in response to alcohol (i.e., neuroplasticity), dependence, relapse, damage to nerve cells (i.e., neurodegeneration), and treatment, which are described in other articles of this issue. In particular, population studies can help researchers understand the neurobiological factors that contribute to patterns of alcohol use, BECs attained, and repeated intoxication.

By identifying individuals who are on the ends of the population spectrum (i.e., who exhibit either particularly low or particularly high daily consumption), investigators can devise experimental designs that address either innate predisposing factors or mechanisms that are activated (i.e., acquired) as a result of ethanol exposure. For example, studies can try to address questions such as whether correlated endophenotypes^7^ (e.g., the degree to which the body’s stress response system—the hypothalamic–pituitary–adrenal [HPA] axis—responds to a stressful situation or the level of neuronal activity in a brain area called the ventral tegmental area) exist in the population and mirror the distribution of drinking.

Most likely, such neurobiological outcomes in humans will only be measured at one time point during an individual’s drinking history (e.g., at the end of chronic drinking); in studies using nonhuman primates and other animal models, however, experimental designs could be devised that address several pivotal points in drinking history. For example, the population distribution of drinking to intoxication (see figure 3) may be substantially different if the animals are retested following repeated withdrawal episodes from ethanol. Thus, it is conceivable that such an imposition of abstinence subsequently will drive the population to a more bimodal rather than a continuous distribution—that is, the animals could fall into two major groups: animals that drink very little and have low BECs and animals that drink a lot and achieve high BECs. Such a change in distribution could indicate that in some individuals drinking is susceptible to “positive” change (i.e., is reduced) following abstinence-based treatments, whereas other individuals may be “adversely” affected (i.e., consume more alcohol) after repeated periods of abstinence.

Population distributions and individual differences in ethanol intake also can help address the genetic basis of the predisposition to drink alcohol to intoxication. The extensive data available on many generations of rhesus monkeys from the National Institutes of Health (NIH) breeding population, which have been tested with a standard protocol of flavored ethanol solutions and short daily sessions, have been used for this purpose (Higley et al. 1991). For example, some investigators have compared the levels of a compound called 5-hydroxyindoleacetic acid (5-HIAA), which is the major breakdown product (i.e., metabolite) of the neurotransmitter serotonin, and alcohol intake in the rhesus monkeys. Some of these studies found a correlation between 5-HIAA levels in the fluid bathing the brain (i.e., cerebrospinal fluid [CSF]) and intake of a sweetened ethanol solution given 1 hour/day, 5 days/week, for 2 to 3 weeks, suggesting that low turnover of serotonin in the brain could be a risk factor for elevated ethanol intake (i.e., 0.8 to 1.4 g/kg) (Barr et al. 2004*a*; Higley et al. 1996*a*,*b*).

The results have not been consistent, however, and in other studies 5-HIAA levels in the CSF did not predict ethanol intake in monkeys obtained from the same breeding population (Fahlke et al. 2002; Vivian et al. 1999). These variations in drinking outcome and CSF 5-HIAA levels potentially may be related to the effects of stressful events early in life on ethanol intake under limited-access conditions. Thus, Bennett and colleagues (2002), who examined both early childhood trauma and ethanol intake, found that a certain gene variant affecting the serotonin system^8^ was associated with lower 5-HIAA levels only under adverse rearing conditions (Bennett et al. 2002). Moreover, the link between the presence of this gene variant (i.e., the genotype) of this transporter molecule and ethanol consumption was significant only for females reared under adverse conditions (Barr et al. 2004*b*). Studies such as these, which are attempting to investigate the association of a specific genotype with physiological/metabolic outcomes (e.g., low 5-HIAA levels in the CSF) as well as alcohol consumption, are likely to become more prevalent in nonhuman primate studies of risk for alcoholism. Clearly, these monkey studies allow researchers to examine both traumatic events and drinking outcomes in the context of stringent experimental approaches (e.g., randomly assigning subjects to different groups) that are not possible in studies on humans.

An important consideration for these animal experiments is, however, to what extent findings in monkeys can be applied to humans. Today, the translation of animal data to human subjects based on analogous genetic influences appears well underway. Indeed, the study of behavioral genetics has begun to uncover intriguing parallels between human and nonhuman primates, including with respect to several genes associated with anxiety, depression, and alcohol consumption, such as:

- A gene called *SLC6A4* that encodes a serotonin transporter;
- A gene called *TPH2* that encodes a protein involved in serotonin synthesis;
- A gene called *MAOA* that encodes an enzyme involved in the metabolism of the neurotransmitters serotonin and norepinephrine;
- A gene called *ORPM1* that encodes a protein (i.e., the μ-opioid receptor) which mediates the actions of signaling substances known as endogenous opioids; and
- A gene called *CRH* that encodes corticotrophin-releasing hormone, which is involved in the body’s stress response.

In each case, variations in these genes (i.e., polymorphisms) have been identified in rhesus macaques that have the same effects on gene function as do the corresponding polymorphisms found in human populations (Barr et al. 2008; Chen et al. 2006; Vallender et al. 2008*a*,*b*; Wendland et al. 2006). Because it is clear that complex interactions between genetic and environmental risk factors contribute to many psychiatric traits, including those related to alcohol use and abuse, the study of primates whose genetic makeup has been well characterized and who have known similarities or differences in their life histories could clarify the interplay of risk factors.

Some recent investigations have begun to unravel genetic and environmental interactions. For example, Rogers and colleagues (2008), who investigated heightened vigilance (i.e., physical orientation to a human intruder) in rhesus macaques, found vigilance to be a largely heritable trait (*h*^2^ = 0.908), with minimal environmental contributions. In contrast, another study (Kraemer et al. 2008) found that fetal alcohol exposure and a polymorphism in the serotonin transporter gene (i.e., a genetic factor) contribute to irritability and heightened stress responsiveness^9^ in young rhesus macaques that had been separated from their mothers (i.e., an environmental factor). In the next few years, the increasing study of the rhesus macaque genome likely will uncover more analogous gene variants in humans and monkeys, providing more opportunities to explore the complexity of genetic and environmental influences on behavioral disorders.

## Conclusions

The recent growth in studies assessing oral ethanol self-administration in non-human primates speaks to the relevance of this model for determining why people drink. The risk for alcoholism is related to both biological (e.g., species, sex, ethanol metabolism, hormonal response to stress, temperament, brain mechanisms of reinforcement, etc.) and environmental risk factors. To adequately characterize the impact of the various risk factors on the development of alcohol use disorders, specific biological risk factors for excessive ethanol drinking will have to be compared using designs in which environmental factors related to the initiation of alcohol use and subsequent access conditions (e.g., ethanol concentration, ethanol availability, concurrent reinforcers, social dynamics, stressors, etc.) are kept consistent. For such studies, nonhuman primates should provide a wealth of information on the complexities of environmental and genetic factors acting alone and in combination to produce alcoholic phenotypes, such as chronic excessive drinking.

Because of their close relationship with humans—both in terms of their genetic makeup and with respect to their developmental stages as well as social and affective behaviors—nonhuman primates ideally can complement approaches using other animal models and laboratory analyses (i.e., in vitro studies) to investigate the mechanisms contributing to alcohol dependence as well as its consequences, particularly those affecting the brain. As described in the following articles in this issue, much already has been learned about the pathways leading to alcohol dependence as well as recovery from the disease. For example, the development of tolerance to alcohol’s effects, both at a cellular and molecular level and at a behavioral level, can lead to alcohol dependence. In addition, researchers have elucidated the role of neuroadaptation in alcohol dependence, from both a pharmacological perspective and a behavioral perspective.

Animal models also have helped clarify the processes in the brain that occur during withdrawal, how they lead to a propensity for relapse, and what role stress plays in these events. An additional area of neuroscience research that incorporates both animal and human studies addresses the detrimental effects of chronic alcohol use on brain structure (i.e., neurodegeneration) as well as the possibility of recovery during abstinence. Although it is important to understand the diverse processes in the brain that are affected by alcohol, it is equally critical to better understand the role that other biological factors (e.g., age, gender, or presence of comorbid disorders) and external influences (e.g., nutrition or drinking patterns) play in modulating a person’s risk for alcohol dependence. The results of animal and human studies addressing these issues are summarized in another article.

Finally, the results of neuroscience research to elucidate alcohol’s interactions with various brain systems can inform treatment research and contribute to advances, particularly in the area of pharmacotherapy. Animal studies are an integral part of all of these aspects of neuroscience research in the alcohol field, and nonhuman primate studies, including the population distribution analyses described in this article, can help researchers to identify the factors most pertinent to the human condition of alcohol dependence.

## Footnotes

1 Primates can be divided into humans and nonhuman primates. The nonhuman primates fall into three groups, including small animals known as prosimians, monkeys, and apes. The most obvious difference between monkeys and apes is that apes (e.g., gorillas, chimpanzees, bono-bos, orangutans, and gibbons) have no tails, whereas most monkeys have visible tails. In addition, most apes are larger in size than monkeys.

2 This is in the same level that is considered intoxication in humans the United States (Greenfield and Kerr 2008) and is the limit for driving in many States.

3 The total number of samples analyzed was 2,880 taken from 48 monkeys.

4 In other words, the 12-month prevalence rate of alcohol abuse or alcohol dependence as defined in the DSM–IV is approximately 5 percent.

5 Reinforcement is a process in which a response or behavior is strengthened based on previous experiences. One can distinguish positive reinforcement, in which presentation of a presumably rewarding stimulus or experience increases the probability that an individual exhibits a certain response, and negative reinforcement, where the probability of a response increases if the response allows the individual to avoid or alleviate a negative experience.

6 Operant responding means that the animal has to complete a certain task (e.g., press a lever) to receive a dose of alcohol during the period of access.

7 An endophenotype is a heritable trait or characteristic that is thought to be an intermediate between a genetic predisposition and a clinical disorder; for example, certain neurobiological characteristics (i.e., certain brain waves) have been noted in people with alcoholism and may be used as endophenotypes to identify people at risk for alcoholism. Endophenotypes are thought to be useful for gene identification under the assumption that they are simpler and closer to the genetic underpinnings of the disorder.

8 This variant (which is known as rh5-HTTLPR) was found in a DNA region controlling the activity of a gene encoding a molecule that transports serotonin back into nerve cells.

9 These characteristics were determined by measuring the levels of certain stress hormones in different situations.
